# A Novel Role for the Adenovirus L4 Region 22K and 33K Proteins in Adeno-Associated Virus Production

**DOI:** 10.1089/hum.2023.146

**Published:** 2024-01-16

**Authors:** Angela Adsero, Brendan Chestnut, Sara Shahnejat-Bushehri, Lalita Sasnoor, Travis McMurphy, Mike Swenor, Ryan Pasquino, Arun Pradhan, Victor Hernandez, Linas Padegimas, David Dismuke

**Affiliations:** ^1^ Molecular Development, Forge Biologics, Grove City, Ohio, USA.; ^2^ Process Development, Forge Biologics, Grove City, Ohio, USA.; ^3^ Chief Technical Officer, Forge Biologics, Grove City, Ohio, USA.

**Keywords:** adeno-associated virus, AAV, adenovirus, L4 33K, L4 22K, E2a

## Abstract

Despite decades of research in adeno-associated virus (AAV) and the role of adenovirus in production, the interplay of AAV and adenovirus is not fully understood. Specific regions of the adenoviral genome containing E1, E2a, E4 open reading frame (ORF), and VA RNA have been demonstrated as necessary for AAV production; however, incorporating these regions into either a producer cell line or subcloning into an Ad helper plasmid may lead to inclusion of neighboring adenoviral sequence or ORFs with unknown function. Because AAV is frequently used in gene therapies, removing excessive adenovirus sequences improves the Ad helper plasmid size and manufacturability, and may lead to safer vectors for patients. Furthermore, deepening our understanding of the helper virus genes required for recombinant AAV (rAAV) production has the potential to increase yields and manufacturability of rAAV for clinical and commercial applications. One region continuously included in various Ad helper plasmid iterations is the adenoviral E2a promoter region that appears to be necessary for E2a expression. Due to the compact nature of viral genomes, the E2a promoter region overlaps with the Hexon Assembly/100K protein and the L4 region. The L4 region, which contains the coding sequences for 22K and 33K proteins, had not been thought to be necessary for AAV production. Through molecular techniques, this study demonstrates that the adenoviral 22K protein is essential for rAAV production in HEK293 cells by triple transfection and that the 33K protein synergistically increases rAAV yield.

## INTRODUCTION

The *Dependovirus* genus of the *Parvoviridae* family are small, single-stranded, nonenveloped viruses that require a helper virus for replication. The representative *Dependovirus* type was initially detected as a contaminant in a preparation of adenovirus and was aptly named adeno-associated virus (AAV).^1^ AAV has a ∼4.7 kbp single-stranded DNA genome with two main ORFs and several minor ORFs that rely on splicing and alternative translation start sites to produce four replication proteins (Rep78, Rep68, Rep52, and Rep40), three structural capsid proteins (VP1, VP2, and VP3), and three nonstructural proteins (AAP, MAAP, and X).^2–6^

The replication of AAV is normally dependent on a helper virus, such as adenovirus or herpesvirus, and this replication dependency is one of the advantageous features that makes AAV an attractive tool for gene therapy vectors. Five adenoviral (Ad) genes have been shown to be necessary for AAV replication when Ad is the helper virus; these Ad genes include E1a, E1b, E2a, E4 ORF6, and VA RNA.^7–12^

These Ad genes can be supplied to the cell for production of AAV vectors either by integration into the producer cell genome, by use of an Ad helper plasmid, or by adenovirus infection. One commonly used system for the generation of recombinant AAV (rAAV) is the triple-transfection system, where E1 genes are provided by an HEK293 cell line that is transfected with three plasmids to provide (1) AAV rep and cap genes, (2) Ad helper genes (E2a, E4, and VA RNA), and (3) an inverted terminal repeat (ITR)-flanked recombinant genome.

There are many Ad helper plasmids widely used within the gene therapy field and the most common designs retain additional adenoviral sequences, possibly from the convenience of utilizing restriction sites in the adenoviral genome to subclone fragments containing E2a, E4 ORFs, and VA RNA into an Ad helper plasmid.^7^ Including unnecessary sequence makes the plasmid substantially larger and could result in the production of immunogenic proteins.^7^ Since gene therapy safety is a high priority, our first Ad helper plasmid design (pEMBR™) excluded sequence encoding the fiber protein, the packaging protein 3 (L1-52K/55K), the peripentonal hexon-associated protein, and part of the hexon-associated precursor protein.

While our initial Ad helper plasmid design removed several extraneous adenovirus elements, we reasoned that portions of the E2a region might also be dispensable. Besides the E2a ORF, the E2a region contains the natural E2a promoters and ORFs for hexon Assembly/100K protein and the L4 region 33K and 22K proteins. In this study, several constitutive and native promoters were evaluated within Ad helper plasmids for E2a expression and AAV production. We also examined the deletion of the ORFs for hexon assembly/100K protein and L4 33K and 22K, which overlap with the published E2a promoter regions.

Our results showed that the removal of E2a promoter regions abrogated AAV production, despite robust E2a expression. The data presented here indicate that AAV production is dependent not only on E1a, E4 ORF6, VA RNA, and E2a but also on the L4 region. We further discovered that the L4 22K protein is indispensable for maintaining AAV production within the HEK293/transient-transfection system, while the L4 33K protein appears to synergistically increase yield. These discoveries allow further reduction of the Ad helper plasmid size.

## MATERIALS AND METHODS

### Cell lines and plasmids

Ignition Cells™, a proprietary HEK293 suspension culture cell line, were grown and maintained in GlutaMAX supplemented animal component-free media (ThermoFisher) with shaking at 37°C, 5.0% CO_2_.

An AAV2/9 pseudotyped plasmid was used to replicate an AAV2 ITR-flanked GFP transgene from a stuffed backbone plasmid and package into an AAV9 capsid.

The pEMBR plasmid served as the origin for all derived designs. pEMBR is a 12,130 bp Ad helper plasmid consisting of an E2a region, which includes an L4 region, an E4 region, and a VA RNA region. All regions were synthesized and assembled *de novo*. The pEMBR plasmid does not contain nucleotide sequence for a fiber protein, the L1-52/55K protein, or a peripentonal hexon-associated protein.

The plasmids 1.2B2, 1.3, 1.4, 1.4B2, 1.5A, 1.8, 1.9, 250Δ, 500Δ, 750Δ, 1 kbpΔ, 1.5 kbpΔ, 2 kbpΔ, and 2.5 kbpΔ were generated by synthesis and cloning into pEMBR (utilizing Genscript services). The 500 bp deletion was generated as only a 498 bp deletion construct to keep the hexon assembly reading frame occurring antiparallel to E2a intact.

The plasmids GEP +1 kbp, GEP, JEP +1 kbp, JEP, GEP +50 bp, GEP +50 bp + Kozak, 1.5 kbpΔ + JEP, and HexonΔ were all generated by site-directed mutagenesis. The plasmids GEP +50 bp, GEP +50 bp + Kozak, and 1.5 kbpΔ + JEP were generated utilizing Genscript services. These plasmids GEP +1 kbp, GEP, JEP +1 kbp, and JEP, and HexonΔ generated by *in vitro* site-directed mutagenesis reactions utilized the corresponding primers (Table 1). The HexonΔ plasmid was mutated to generate a stop codon at amino acid C32 for hexon assembly, while generating a silent mutation within E2a. Candidates were identified by diagnostic restriction digest.

**Table 1.** Primers and probes

Primer Name	Primer Sequence (5′-3′)	Primer Purpose
GEP +1 kbp	CTCGTTGAAGGCCGTCCATAGGTCCTTCAAGGGCAGGCTCGCGCGTTTGAAGCCAGCGCGCTAG	Site-directed mutagenesis
GEP	GGTTTCGCGCTGCTCCTCTTCCCGACTGGCCATCTTGAAGGACCTATGGACGGCCTTCAACGAG	Site-directed mutagenesis
JEP +1 kbp	GCGCGCTGGCTTCAAACGCGCGAGCCTGCCAAGCGAAGATCAGCTTCGGCGCACGCTGGAAG	Site-directed mutagenesis
JEP	GGTTTCGCGCTGCTCCTCTTCCCGACTGGCCATAAGCGAAGATCAGCTTCGGCGCACGCTGGAAG	Site-directed mutagenesis
HexonΔ	GCGCGTGGTATGCGGACTCAAGAGGAAGAGGAAGAGCC	Site-directed mutagenesis
eGFP forward	GAACCGCATCGAGCTGAA	qPCR primer
eGFP reverse	TGCTTGTCGGCCATGATATAG	qPCR primer
eGFP probe	FAM-atcgacttcaaggaggacggcaac-3′-Iowa Black™ FQ	qPCR probe

qPCR, quantitative real-time PCR.

The pUC57 vectors used in quadruple transfection were generated utilizing Genscript services to synthesize the expression cassette, clone into their pUC57 backbone, and provide plasmid. The L4 expression cassettes previously tested in trans were utilized in subcloning to produce an Ef1α-driven codon-optimized 22K. Then Ef1α-driven L4/22K expression cassettes were subcloned into the plasmid backbones indicated in the text.

Plasmid prepped in-house utilized Promega PureYield Plasmid Miniprep System, Promega PureYield Maxiprep System, or ThermoFisher PureLink HiPure Plasmid Maxiprep Kit. Plasmid production for plasmid cloned in-house was also outsourced to Genscript and Aldevron. All plasmids were sequenced, verified by Genscript or in-house services.

### AAV vector production

Ignition cells, a proprietary HEK293 suspension culture cell line, were transfected with the Ad helper plasmid(s), the rep cap plasmid, and the ITR-flanked GFP transgene plasmid using a PEI method. Triple transfection utilized one Ad helper plasmid, while quadruple transfection utilized Ad helper plasmids. Triple transfected cells were transfected with 1.0 pg of DNA per cell utilizing linear PEI (Polysciences). Ninety-six hours post-transfection, 1 mL of culture was removed to generate radioimmunoprecipitation assay (RIPA) whole cell lysate for Western blotting. The remaining culture was triton lysed by 4-h shaking incubation at 37°C. Lysate was centrifuged at 4,000*g* for 15 min at 4°C and a 0.2 μm filter.

### Titer determination

AAV titer was determined using quantitative real-time PCR (qPCR) with a QuantStudio™ 6/7. Negative and positive controls were used in each run. All diluted samples were tested in triplicate. Reaction mixtures contained 1 × TaqMan Fast Advanced Master Mix (Applied Biosystems), 600 nM of primers, and 300 nM of probe to eGFP (IDT; Table 1). The standard curve was generated using seven 1:10 serial dilutions of an eGFP DNA gblock (IDT).

### Western blotting

RIPA whole cell lysate was prepared by RIPA buffer (ThermoFisher catalog#89900) lysis with Halt™ protease inhibitor (ThermoFisher catalog#1861278). Lysate supernatant was harvested after a 15-min 18,000*g* centrifugation. The ThermoFisher/Invitrogen Bolt Bis-Tris Plus system was used to separate equal amounts of total protein (determined from a Bio-Rad DC protein assay, catalog#5000112) on a gradient 4–12% gel. Separated protein was transferred to a polyvinylidene fluoride membrane using the ThermoFisher/Invitrogen iBlot2 dry blotting system at 15 V for 7 min. Ponceau stain was used to verify even loading. Blocking occurred for 1 h in tris-buffered saline with tween or phosphate-buffered saline with tween buffer with either 5% nonfat dry milk or bovine serum albumin.

Antibody diluent mirrored the blocking buffer. Primary antibodies for E2a (Cusabio rabbit DBP antibody catalog#CSB-PA365892ZA01HIL; 1:2,000), Rep (Progen mouse anti-Rep catalog#61069; 1:100), and β-actin (Abcam Rabbit Beta-Actin catalog#ab8227; 1:2,000) were all used overnight at 4°C. Secondary antibodies were horseradish peroxidase-conjugated goat anti-rabbit (Abcam catalog#ab205718; 1:40,000) and rabbit anti-mouse (Abcam catalog#ab97046; 1:10,000) incubated for 1 h at room temperature. Blots were developed using the ThermoFisher SuperSignal West Pico PLUS substrate (catalog#34580) and imaged by an Amersham ImageQuant800 system.

## RESULTS

### Numerous E2a constructs with AAV production loss demonstrate robust E2a expression

**Figure 1.**
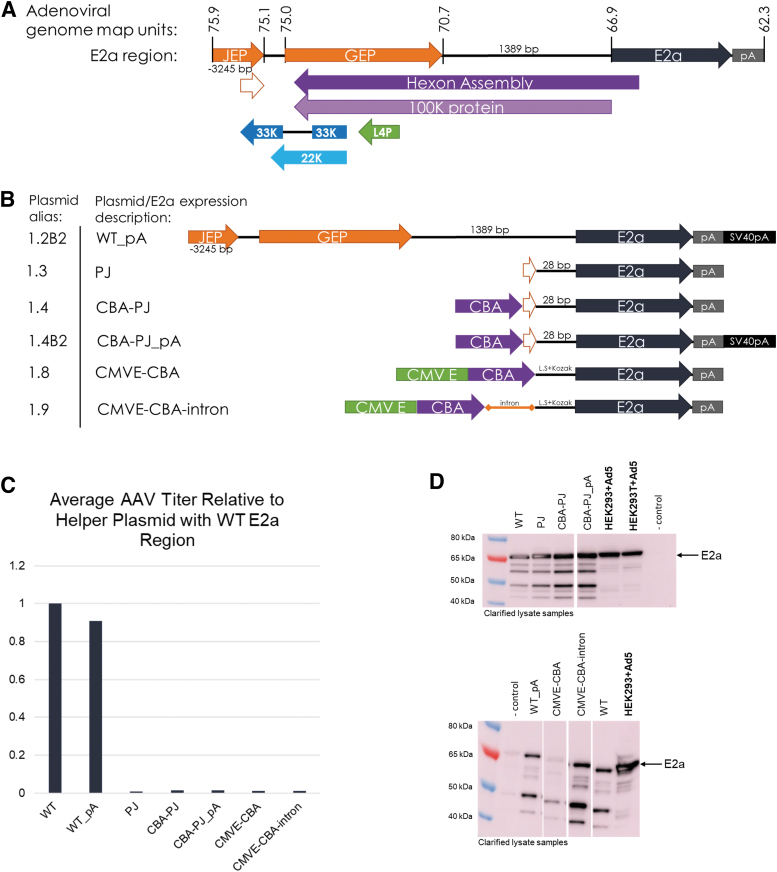
Production of rAAV is abrogated when 5′ E2a region is modified, despite robust E2a expression. **(A)** The adenoviral type 5 genome (human mastadenovirus C serotype 5 AY339865.1) from 22394 to 27277 base pairs (bp; shown in map units) was used as the WT E2a region. The corresponding regions for the Ad2 E2a promoters, GEP and JEP, are indicated. The core E2a promoter, PJ, sequence is depicted by the smallest open arrow and corresponds to the Ad5 genome map units 75.6–75.1. This WT E2a region also contains ORFs for hexon assembly/100K protein and the L4 region 33K and 22K proteins. **(B)** Numerous E2a expression cassettes utilized in the Ad helper plasmid for rAAV production by triple transfection of Ignition Cells™ (HEK293 suspension) are depicted. Exogenous elements added may include CBA promoter, CMVE, a chimeric intron (intron), and simian virus polyadenylation site (SV40 polyA). The length (bp) of the native E2a upstream sequence is given, except for those that contain 25 bp of the native upstream sequence with a Kozak sequence added (LS + Kozak). **(C)** The average titer of rAAV9 from triple transfection clarified lysate (harvest) samples determined by qPCR is graphed relative to the Ad helper containing the WT E2a region for the E2a constructs described in **(B)**. **(D)** E2a Western blot of the clarified lysate samples from **(C)**. Adenovirus type 5-infected HEK293/HEK293T cells are shown as a positive control and uninfected/nontransfected HEK293 cells are shown as a negative control. CBA, chicken beta actin; CMVE, cytomegalovirus immediate early enhancer; LS, leader sequence; ORF, open reading frame; PJ, partial JEP; qPCR, quantitative real-time PCR; rAAV, recombinant AAV; WT, wild type.

The E2a region that is found in adenovirus and within many commercially available Ad helper plasmids includes ∼3,500 bp of upstream sequence to the E2a ORF. We mapped the Ad2 E2a promoters identified by several groups on the human mastadenovirus C serotype 5 (Ad5) genome (GeneBank AY339865.1) and in our pEMBR helper plasmid (Fig. 1A, B).^13–15^ “JEP” corresponds to the Jing et al.-identified E2a Promoter and “GEP” corresponds to the late E2a expression Guilfoyle et al.-identified E2a Promoter (Fig. 1A, B). The core E2a promoter (partial JEP/PJ) is contained within JEP and was identified by Casper et al.^14^ These E2a promoter regions also overlap with the adenovirus hexon assembly/100K protein and L4 coding regions.

To further minimize potential immunogenic sequences in the pEMBR Ad helper plasmid and reduce overall plasmid size, we created several Ad helper plasmids containing different E2a expression cassettes to replace most of the E2a upstream sequence with elements aimed at maintaining high E2a expression (Fig. 1B). The designed E2a expression cassettes removed the hexon assembly/100K protein and L4 coding regions within the pEMBR helper plasmid and replaced the E2a promoter function with the chicken beta actin (CBA) promoter, with or without a human cytomegalovirus enhancer or a chimeric intron.

Triple transfection of HEK293 cells was performed to test these new Ad helper plasmids for AAV production in combination with an AAV9 rep/cap plasmid and an ITR-flanked GFP transgene plasmid. All E2a constructs missing the full upstream wild-type (WT) sequence were unable to produce rAAV above the limit of quantitation (LOQ) assayed by qPCR (Fig. 1C). Clarified lysate samples were analyzed by Western blot for E2a protein expression to verify that the selected E2a promoter region was functional. We found that all Ad helper plasmids with PJ-driven E2a had similar E2a expression compared to the WT E2a region (Fig. 1D, top panel). The decrease in E2a expression observed using the cytomegalovirus immediate early enhancer-CBA promoter appeared to be rescued by the addition of a chimeric intron (Fig. 1D, bottom panel).

### Upstream E2a adenoviral sequence removal was explored with internal deletions, truncations, and mutations

While E2a expression appeared adequate, it was possible that a regulatory region was impacted and this affected E2a expression kinetics. In this light, internal deletion constructs were designed to study the native promoter effects on rAAV production and E2a expression. In parallel, we also added an SV40 polyadenylation sequence to the adjacent E4 region in the plasmid to ensure that E2a region changes did not affect the adjacent E4 region (data not shown).

**Figure 2.**
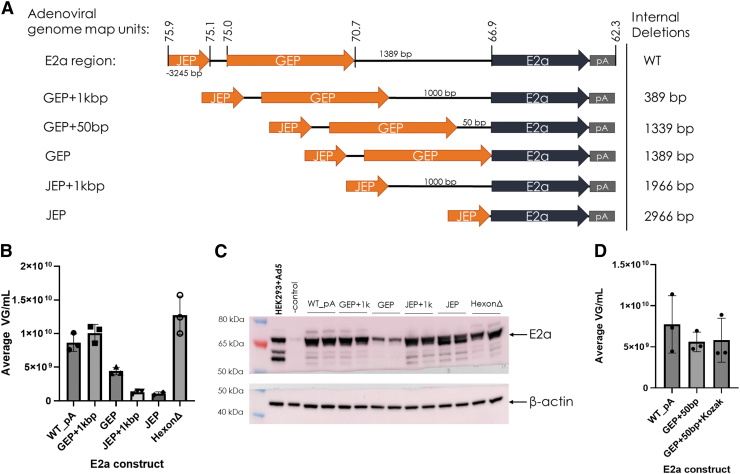
Systematic deletions in 5′ E2a region distinguish regions required for E2a expression and rAAV production. **(A)** E2a expression cassettes with internal region deletions utilized in the Ad helper plasmid for triple transfection of Ignition Cells (HEK293 suspension). The same adenoviral type 5 genome is shown as the reference WT E2a region as in Fig. 1A. The corresponding regions for GEP and JEP are indicated. Constructs containing both JEP and GEP regions are named GEP, whereas constructs containing only JEP are named JEP. These internal deletion constructs were generated by site-directed mutagenesis. **(B)** Average rAAV titer (VG/mL) from triplicate (except JEP performed in duplicate) 500 mL triple transfected cultures determined by qPCR for the constructs described in **(A),** as well as the hexon assembly deletion mutant (HexonΔ). **(C)** E2a Western blot of RIPA lysates from the transfected cultures in **(B)**. Each E2a construct was analyzed from duplicate samples. Beta-actin is shown as a loading control. Adenovirus type 5-infected HEK293/HEK293T cells are shown as a positive control and uninfected/nontransfected HEK293 cells are shown as a negative control. **(D)** Average rAAV titer (VG/mL) from triplicate 75 mL triple transfected cultures determined by qPCR for the GEP +50 bp and GEP +50 bp + Kozak constructs in comparison to WT (WT_pA). RIPA, radioimmunoprecipitation assay; VG, vector genomes.

The Ad helper plasmids featuring internal deletions to investigate the native promoters were named according to the proximal promoter and amount of native E2a leader sequence (Fig. 2A). Along with these native promoter variations, we also tested a hexon assembly deletion mutant (hexonΔ) to verify that altering this ORF in initial designs did not cause production failure.

Due to hexon assembly/100K's overlapping antiparallel ORF to E2a, we used site-directed mutagenesis to introduce a silent mutation within E2a and generate a stop codon within hexon assembly. AAV production was comparable to WT (WT_PA) with both the GEP +1 kbp and hexonΔ Ad helper plasmids (Fig. 2B). Because the GEP +1 kbp plasmid features an internal 389 bp deletion, which also affects the hexon assembly/100K ORF, we obtained two indications that hexon assembly/100K is dispensable. Simultaneous testing of a 100K (truncated hexon assembly) protein deletion also resulted in no effect on production (data not shown).

Surprisingly, JEP Ad helper plasmids did not support rAAV production, despite a high level of E2a expression regardless of upstream sequence inclusion/exclusion (Fig. 2B, C). Upstream sequence inclusion did have an effect when both GEP and JEP were present, because the GEP Ad helper had reduced E2a expression with a correlative reduction in yield when compared to WT and GEP +1 kbp (Fig. 2B, C). To add a short leader to the GEP plasmid, Ad helpers were generated to include 50 bp of upstream E2a sequence (GEP +50 bp) as well as adding a Kozak sequence (GEP +50 bp + Kozak; Fig. 2A). These Ad helper plasmids produced rAAV similar to WT (WT_PA; Fig. 2D). These results supported the removal of 1,339 bp between GEP and E2a to reduce adenoviral sequence and Ad helper plasmid size.

**Figure 3.**
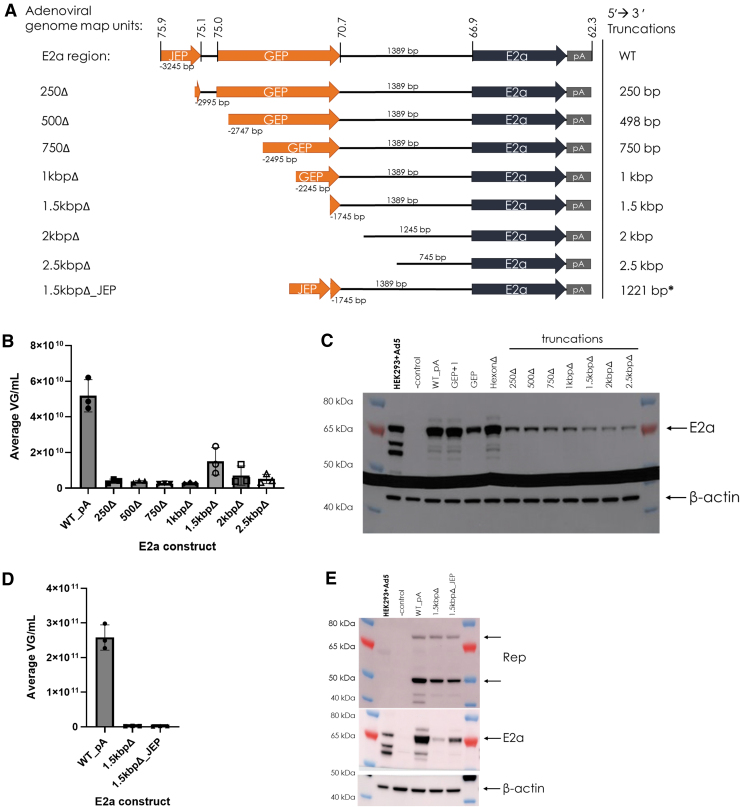
5′→3′ truncations decrease E2a expression and eliminate AAV production; JEP addition increases E2a expression, but does not restore AAV production. **(A)** The same WT E2a region is shown as depicted in Fig. 1A. These additional E2a constructs were synthesized to contain a 5′→3′ truncation of the region as named and were cloned into the helper plasmid. **(B)** Average rAAV titer (VG/mL) from triplicate 75 mL triple transfected cultures determined by qPCR for the truncated E2a constructs in comparison to WT (WT_pA). **(C)** E2a Western blot of RIPA lysates from transfected cultures in B. Samples from Fig. 2C were used as controls. Beta-actin is shown as a loading control. Adenovirus type 5-infected HEK293/HEK293T cells are shown as a positive control and uninfected/nontransfected HEK293 cells are shown as a negative control. **(D)** Average rAAV titer (VG/mL) from the triplicate 75 mL triple transfected cultures determined by qPCR for the 1.5 kbpΔ constructs in comparison to WT (WT_pA). **(E)** Rep and E2a Western blots of RIPA lysates from the transfected cultures in **(D)**. Beta-actin is shown as a loading control.

To further define the E2a promoter region, a series of 5′→3′ truncations of the E2a region were tested (Fig. 3). Almost all truncated E2a region Ad helper plasmids did not support rAAV production above the LOQ by qPCR (Fig. 3B). A dramatic decrease in E2a expression was observed with the first truncation and continued to decrease with subsequent deletions until no E2a promoter sequence remained (Fig. 3C). Since the 1.5 kbpΔ construct initially appeared to have low rAAV production, another Ad helper plasmid was generated to add JEP to the 1.5 kbpΔ construct (1.5 kbpΔ_JEP, a 1,221 bp internal deletion from WT_PA) to test if increasing E2a expression would increase production (Fig. 3A). Even though E2a expression increased, rAAV production did not increase (Fig. 3D, E).

### AAV production is rescued by the L4 region 33K and 22K coding sequence

**Figure 4.**
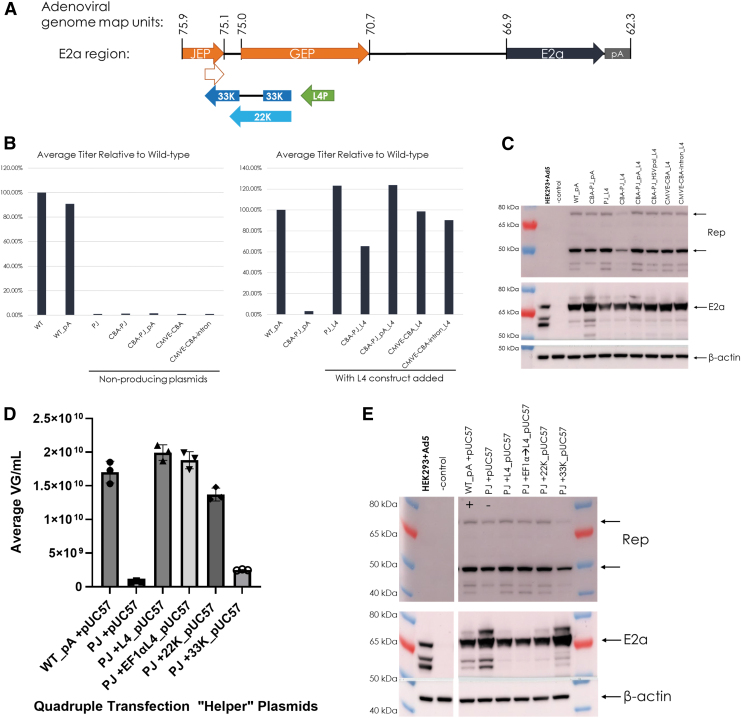
rAAV production is rescued when the L4 region (coding for Ad 33K and 22K proteins) is provided *in cis* or *in trans*. **(A)** The Fig. 1A WT E2a region is shown with the additional depiction of the L4 region ORFs for 22K and 33K protein. The PJ sequence is depicted below JEP by the *smallest open arrow*, to draw attention to how this natural E2a promoter sequence will be added into every construct when the L4 33K protein is added. **(B)** The graph from Fig. 1B (*left*) to compare with the production rescue (*right*) observed when the L4 cassette is added (denoted by “_L4”) to the plasmids described in Fig. 1A. The L4 cassette contains the natural promoter, the 33K and 22K ORFs, and terminates with a 49 bp synthetic polyA tail. The average titer of rAAV9 from triplicate 75 mL triple transfected cultures determined by qPCR was graphed relative to the helper containing the WT E2a region (WT_pA). **(C)** Rep and E2a Western blots of RIPA lysates from the transfected cultures from **(B)** (*right*). Beta-actin is shown as a loading control. Adenovirus type 5-infected HEK293/HEK293T cells are shown as a positive control and uninfected/nontransfected HEK293 cells are shown as a negative control. **(D)** Average rAAV titer (VG/mL) from triplicate 75 mL quadruple plasmid transfected cultures determined by qPCR for the PJ E2a construct plus the pUC57 vector containing the specified L4 construct in comparison to WT plus empty vector (WT_pA + pUC57) production. The PJ construct is shown as a reference. **(E)** Rep and E2a Western blots of RIPA lysates from the transfected cultures in **(A)**. The same loading, positive, and negative controls as C were used.

The data from truncating or replacing the upstream E2a region suggested that factors within this region are necessary for rAAV production and are independent from E2a expression, since high E2a protein levels could be obtained. Production was only maintained with the entire JEP-GEP region, which also encodes the L4 region 33K and 22K proteins (Fig. 4A).

An L4 expression cassette was designed to include the native L4 promoter, the coding region for 33K and by nature 22K, and a 49 bp synthetic polyadenylation sequence. This L4 expression cassette was cloned upstream of the E2a expression cassette in the initial nonproducing Ad helper plasmids (Fig. 1A). Due to sequence overlap, inclusion of the 33K ORF also adds a PJ that could serve as an additional E2a promoter (Fig. 4A). The L4 expression cassette restored rAAV production of initial designs (compared to WT_PA; Fig. 4B; refer to Fig. 1B for initial designs). The CBA-PJ Ad helper plasmid had an incomplete rescue, but this could be attributed to the observed lower expression of Rep proteins (Fig. 4B, C). All Ad helper plasmids had similar E2a expression (Fig. 4C).

To prove production rescue by the L4 region was due to the 33K and 22K ORFs and not *in cis* regulation of E2a, a quadruple transfection was performed to add the L4 region construct *in trans* from the previously nonproducing Ad helper plasmid, containing the PJ promoter. Adding the L4 region *in trans* restored rAAV production regardless of whether the native promoter-driven L4 (L4_pUC57) or an exogenously driven (by human elongation factor 1 alpha, Ef1αL4_pUC57) L4 was added (Fig. 4D). To determine if either L4 protein was more essential, we also utilized plasmids with natively driven codon-optimized 22K or 33K (22K_pUC57 and 33K_pUC57). Our results indicated that 22K was more essential to rAAV production; however, it appeared that the L4 region proteins' effects on production were additive (Fig. 4D). Analysis of E2a protein by Western blot revealed a slight decrease in E2a protein when 22K was present (Fig. 4E).

### The L4 region can be modified to reduce potential influence on E2a protein expression

To further reduce the size of the Ad helper plasmid and to decrease the potential influence that the upstream L4 region sequence may have on E2a expression, new Ad helpers were generated by cloning L4 expression cassettes into the previously nonproducing PJ and CBA-PJ_pA plasmid backbones and tested for AAV production.

**Figure 5.**
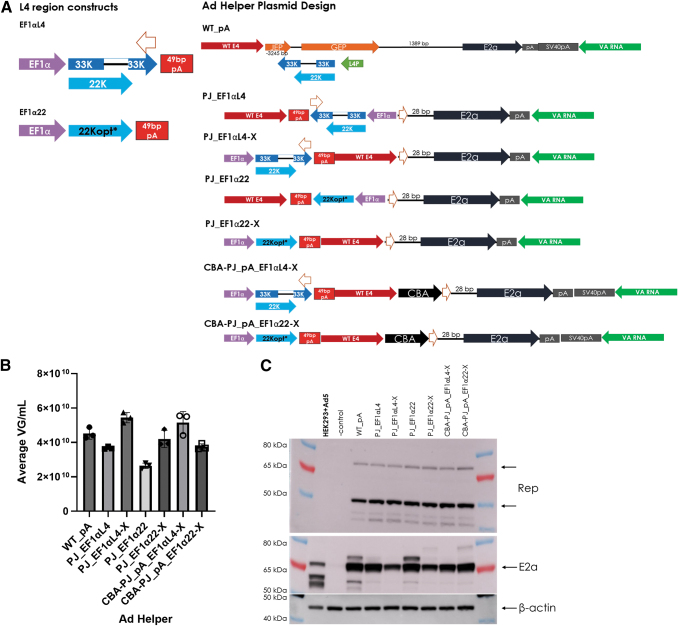
The L4 region is modified for *in cis* L4 (33K and 22K) or 22K expression to reduce potential E2a regulation. **(A)** The L4 region constructs were incorporated into the indicated locations of the previous nonproducing plasmids PJ and CBA-PJ_pA. The EF1αL4 cassette contains the Ef1α promoter, the 33K and 22K ORFs, and terminates with a 49 bp synthetic polyA tail. The EF1α22 cassette contains the Ef1α promoter, the codon-optimized 22K ORF, and terminates with a 49 bp synthetic polyA tail. **(B)** The average rAAV titer (VG/mL) from triplicate 75 mL triple plasmid transfected cultures using the specified Ad Helper shown in **(A)** or the wild-type (WT_pA) Ad Helper plasmid was determined by qPCR. **(C)** Rep and E2a Western blots of RIPA lysates from the transfected cultures in **(B)**. Beta-actin is shown as a loading control. Adenovirus type 5-infected HEK293/HEK293T cells are shown as a positive control and uninfected/nontransfected HEK293 cells are shown as a negative control.

Several design changes were made to achieve this aim. First, the native L4/22K promoter was replaced with the Ef1α core promoter (named Ef1αL4 for 22K and 33K protein expression or Ef1α22 for only 22K protein expression). Second, the 22K codon-optimized sequence was utilized to reduce homology to the native L4 region sequence (a feature of all plasmids that include the Ef1α22 cassette). The third and last change was testing the Ef1α-driven L4/22K cassette in a different plasmid location to prevent *in cis* regulation of E2a, which is indicated in the name by addition of “-X” (Fig. 5A). All tested designs produced AAV (Fig. 5B), providing further confirmation that the L4 region contribution to AAV production can be decoupled from E2a promoter activity.

These data also indicated that the titer might be boosted by inclusion of 33K; however, moving the L4/22K expression cassettes to a different location appeared to have the best effect on titer. Curiously, E2a protein expression was affected by the L4/22K location change in which expression of E2a was decreased when the L4/22K cassette was relocated to prevent *in cis* regulation of E2a (Fig. 5C). This effect is only apparent in the PJ Ad helper plasmid background, since the CBA-PJ_PA background did not have an *in situ* control for comparison (Fig. 5C). This observed effect on E2a protein was similar to the effect observed with *in trans* L4/22K addition, when the L4 region sequence was also unable to provide *in cis* regulation of E2a from the overlapping GEP promoter.

## DISCUSSION

The AAV-Ad interaction has been extensively studied, leading to the conclusion that five adenoviral genes were required for AAV replication and production: E1a, E1b, E2a, E4ORF6, and VA RNA. In our efforts to reduce the Ad helper plasmid, we made the surprising discovery that a sixth factor, the 22K protein, is also required for rAAV production. Our findings show that 22K absence resulted in rAAV production loss. The 22K requirement was further demonstrated when a 22K expression cassette provided *in trans* rescued rAAV production (Fig. 4D).

We also observe a 22K-influenced decrease on E2a expression, but only in cases when the L4 region serves as a protein encoding region and cannot function as an additional E2a regulatory region (Figs. 4E and 5C). This complex E2a regulatory function is likely a minor role for 22K, especially when there appears to be an E2a protein threshold for WT level AAV yields (GEP vs. GEP +1 kbp; Fig. 2B, C). The clarification of Ad genes required for rAAV production and discovery of 22K's essential role in this process is a substantial advancement in understanding the Ad-AAV interaction that could improve rAAV manufacturing.

The reduction in size of the Ad helper plasmid is another rationale for why this work is important. By taking a molecular approach to understanding what sequences are necessary for AAV production, the overall plasmid size can be reduced; these smaller Ad helper plasmids may improve manufacturability and thereby reduce plasmid manufacturing costs. By showing that 22K is the most important L4 protein in AAV production, there is the possibility of further reducing adenovirus sequence within the Ad helper plasmid potentially by removing the 33K C-terminal sequence (301 bp) and replacing the L4 promoter region (187 bp) to reduce plasmid size (Fig. 5B). However, since our results indicated that AAV yield may be diminished without 33K (Figs. 4D and 5B), the AAV titer loss may not justify the minimal decrease in plasmid size.

Our studies support a previously unidentified role for the Ad 33K protein since 33K appeared to synergistically boost AAV production (Figs. 4D and 5B). A 33K-dependent influence on Rep expression was also observed (Fig. 4E). It is unclear at this time how these observations fit into AAV-Ad interactions and more work is needed to tease out a mechanism.

However, both L4 proteins are implicated in adenovirus packaging through interactions with empty capsid, E2a, and the ATPase, IVa2, so it is reasonable to propose that they may have a similar function during AAV production where 22K is required and 33K might increase efficiency.^16–19^ Even though the HEK293 cell line contains a partial IVa2 ORF, which may support adenovirus viability, IVa2-facilitated AAV packaging is questionable since IVa2 appears to specifically bind the Ad packaging domain.^19–22^ Instead, the IVa2 role(s) might be substituted with AAV Rep protein(s) to specifically bind the AAV ITR/packaging sequence.

Interestingly, another group recently noted L4 protein involvement in AAV production, although in an adenovirus-dependent production system. This information was presented at the ASGCT 2023 conference by Su, Cawood, and Seymour, where they demonstrated L4 region proteins have a regulatory-like role on AAV production.^23^

Their data suggested that the L4 22K–33K unit is needed to amplify the integrated rep-cap genes from stable cell lines to produce enough Rep and Cap proteins for rAAV replication using their “Tetracycline-Enabled Self-Silencing Adenovirus” or TESSA system. Rep-cap amplification could be recovered when TESSA genome replication was impaired, rep and cap genes were provided *in trans*, or in their TESSA 2.0 system where rAAV and rep-cap replication were coupled to TESSA genome replication.^23,24^ This regulatory-like role cannot explain the production requirement for the L4 proteins in our system when rep-cap is provided *in trans*, nor does it clearly separate the role of L4 proteins in Ad replication from rAAV production. This inconsistency suggests additional avenues to explore in adenoviral-AAV interactions.

We also define the minimal promoter region needed for WT level E2a expression in the Ad5-AAV system is the PJ or the corresponding core promoter region determined for Ad2 (all Figures).^14^ The GEP region could not exclusively maintain WT level E2a expression, as evidenced with the E2a truncation constructs (Fig. 3). While we recognize that the truncations affect the L4 region, we have shown to be important for AAV production; in this case, production loss might also be attributed to inadequate E2a expression.

Therefore, the L4 region DNA sequence functions as both an E2a promoter and a protein-encoding sequence, whereas the sequence remaining between L4 and E2a is dispensable. This dispensable sequence would also include a substantial portion of hexon assembly/100K protein. Our work suggests it is highly unlikely that hexon assembly/100K contributes to AAV production, since we do not observe production changes with the mutations and deletions tested in hexon assembly/100K that should inhibit expression of these proteins (Fig. 2, see text). Due to the overlapping nature of hexon assembly/100K with the L4 region and E2a region, we were unable to directly demonstrate hexon assembly or 100K protein loss. Future experiments may better address our suggested nonessential roles for hexon assembly and 100K protein.

While our findings shed light on the Ad 22K and 33K genes' roles in rAAV production, further investigations are needed to fully elucidate the mechanistic details of how the 22K and 33K proteins might be facilitating AAV replication and production. More research is required to optimize 22K and 33K use in rAAV production strategies. This future work will help maximize the potential benefits of this discovery in the field of gene therapy.
